# Rapid nosocomial spread of SARS-CoV-2 in a French geriatric unit

**DOI:** 10.1017/ice.2020.99

**Published:** 2020-03-30

**Authors:** Philippe Vanhems, Mitra Saadatian-Elahi, Michel Chuzeville, Elodie Marion, Louise Favrelle, Delphine Hilliquin, Geraldine Martin-Gaujard, Robin Gourmelon, Mathilde Noaillon, Nagham Khanafer

**Affiliations:** 1 Service d'Hygiène, Epidémiologie et Prévention, Hôpital Edouard Herriot, Hospices Civils de Lyon, Lyon, France; 2 Laboratoire des Pathogènes Emergents - Fondation Mérieux, Centre International de Recherche en Infectiologie, Institut National de la Santé et de la Recherche Médicale U1111, Centre National de la Recherche Scientifique, UMR5308, Ecole Normale Supérieure de Lyon, Université Claude Bernard Lyon 1, 21, Avenue Tony Garnier, 69007 Lyon, France; 3 Service de Gériatrie, Hôpital Edouard Herriot, Hospices Civils de Lyon, Lyon, France

*To the Editor*—SARS-CoV2 nosocomial transmission has been reported among healthcare professionals and patients.^1^ However, few studies have focused on nosocomial clusters in elderly patients at high risk of morbidity and mortality.^1^

With >6,600 cases, France is the fourth most affected European country. Edouard Herriot University Hospital (1,100 beds) is the largest emergency hospital in the Lyon area. We report the extremely rapid spread of COVID-19 in a 24-bed geriatric unit.

Epidemiological investigation revealed the existence of 2 potential index cases. The first was a 97-year-old male admitted to the emergency room (ER) with fever and dyspnea on February 29. The nasal swab for influenza and respiratory syncytial virus collected the same day was negative by polymerase chain reaction assay (PCR). The patient was transferred to the geriatric ward without complementary precautions. A second nasal swab was collected on March 7 and was positive for SARS-CoV2 by reverse-transcriptase PCR (RT-PCR). The second potential index case was a 76-year-old man admitted to the ER with cough and fever on February 1. Infection control measures were set up and nasal swab for influenza and respiratory syncytial virus (RSV) was negative by PCR. On March 3, the patient was transferred to the geriatric ward, where preventive air and contact measures were in place. The nasal swab previously collected was retested on March 6 and confirmed positive for SARS-CoV2 by RT-PCR.

**Fig. 1.**
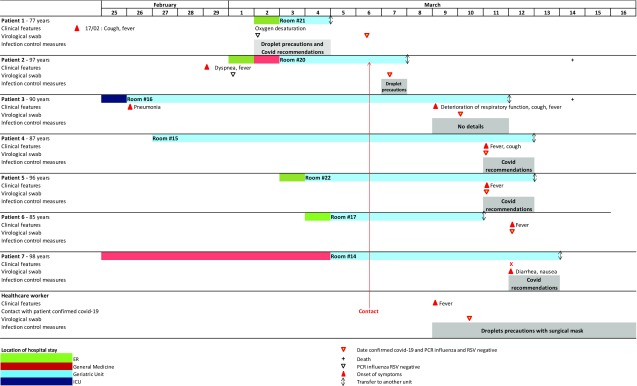
Timeline of exposures and onset nosocomial COVID-19 cases in a geriatric unit, Lyon, France.

The first secondary case of COVID-19 was diagnosed on March 10, and 5 other cases (including a medical doctor) occurred in the same unit until March 13 (Fig. 1). Strict infection control measures and close monitoring of suspected cases of patients and healthcare professionals were subsequently performed to contain the intraunit transmission of the SARS-Cov-2 virus. The infection rate among patients was 20%. Two patients (28.6%) died on March 14. No additional cases occurred.

The likelihood of other sources of infection remains low, and no cases occurred in other areas of the ward. The area where the cases occurred was not primarily selected for COVID-19 hospitalizations, and only 123 cases had been reported to the Lyon Regional Health Agency as of March 14, for a metropolitan area of 2,300,000 inhabitants.

The rapid spread of nosocomial COVID-19 in this ward confirms the contagiousness of SARS-CoV-2 in healthcare settings and the high mortality rates in this population. The existence of super-shedders has been suggested,^2,3^ which could facilitate cluster emergence.

We wish to stress the urgency of strict application of COVID-19 infection control guidelines in healthcare facilities, particularly in geriatric units.
